# Dormant and after-Ripened *Arabidopsis thaliana* Seeds are Distinguished by Early Transcriptional Differences in the Imbibed State

**DOI:** 10.3389/fpls.2016.01323

**Published:** 2016-08-30

**Authors:** Bas J. W. Dekkers, Simon P. Pearce, R. P. M. van Bolderen-Veldkamp, Michael J. Holdsworth, Leónie Bentsink

**Affiliations:** ^1^Department of Molecular Plant Physiology, Utrecht UniversityUtrecht, Netherlands; ^2^Wageningen Seed Laboratory, Laboratory of Plant Physiology, Wageningen UniversityWageningen, Netherlands; ^3^Faculty of Biology, Medicine and Health, University of ManchesterManchester, UK; ^4^School of Mathematics, University of ManchesterManchester, UK; ^5^Division of Plant and Crop Science, School of Biosciences, University of NottinghamLeicestershire, UK

**Keywords:** after-ripening, *Arabidopsis*, dormancy, embryo, endosperm, seed, transcriptome

## Abstract

Seed dormancy is a genetically controlled block preventing the germination of imbibed seeds in favorable conditions. It requires a period of dry storage (after-ripening) or certain environmental conditions to be overcome. Dormancy is an important seed trait, which is under selective pressure, to control the seasonal timing of seed germination. Dormant and non-dormant (after-ripened) seeds are characterized by large sets of differentially expressed genes. However, little information is available concerning the temporal and spatial transcriptional changes during early stages of rehydration in dormant and non-dormant seeds. We employed genome-wide transcriptome analysis on seeds of the model plant *Arabidopsis thaliana* to investigate transcriptional changes in dry seeds upon rehydration. We analyzed gene expression of dormant and after-ripened seeds of the Cvi accession over four time points and two seed compartments (the embryo and surrounding single cell layer endosperm), during the first 24 h after sowing. This work provides a global view of gene expression changes in dormant and non-dormant seeds with temporal and spatial detail, and these may be visualized via a web accessible tool (http://www.wageningenseedlab.nl/resources). A large proportion of transcripts change similarly in both dormant and non-dormant seeds upon rehydration, however, the first differences in transcript abundances become visible shortly after the initiation of imbibition, indicating that changes induced by after-ripening are detected and responded to rapidly upon rehydration. We identified several gene expression profiles which contribute to differential gene expression between dormant and non-dormant samples. Genes with enhanced expression in the endosperm of dormant seeds were overrepresented for stress-related Gene Ontology categories, suggesting a protective role for the endosperm against biotic and abiotic stress to support persistence of the dormant seed in its environment.

## Introduction

Seeds are complex, stress-resistant plant structures with two major functions: reproduction and dispersal. For survival of a species, it is critical that its offspring germinates at the right time of year, and seed dormancy is the trait that helps to achieve this. Seed dormancy is defined as a temporary inability of a viable seed to complete germination under normally favorable conditions (Bewley, 1997). The most prevalent form of seed dormancy is physiological dormancy (Finch-Savage and Leubner-Metzger, 2006), an endogenous block that is present in the embryo (embryo dormancy) and/or in the surrounding layers (endosperm/testa, coat enhanced dormancy). In *Arabidopsis*, seed dormancy is classified as ‘non-deep physiological dormancy’ [in common with seeds of most weeds, vegetables, and many garden flowers (Baskin and Baskin, 2014)] and is usually broken by relatively short periods of cold stratification or dry seed storage at room temperature (a process known as after-ripening; Finch-Savage and Leubner-Metzger, 2006; Bewley et al., 2013; Baskin and Baskin, 2014).

Dormancy is regulated by an intricate web of plant hormone interactions in which the balance between two hormones, abscisic acid (ABA, required for dormancy induction) and gibberellins (GA, required for germination), is of particular importance (Koornneef and van der Veen, 1980; Koornneef et al., 1982; Ogawa et al., 2003; Kucera et al., 2005; Nambara and Marion-Poll, 2005; Finch-Savage and Leubner-Metzger, 2006; Finkelstein et al., 2008). The depth of dormancy in fully matured seeds is determined both by environmental conditions, particularly temperature, experienced by the mother plant (Fenner, 1991; Kendall and Penfield, 2012; Chen et al., 2014; He et al., 2014) as well as genetic factors (Bentsink et al., 2007; Bentsink and Koornneef, 2008). Genetic variation for seed dormancy has been explored by genome wide association studies, as well as quantitative trait loci (QTL) mapping (Alonso-Blanco et al., 2003; Bentsink et al., 2010; Yano et al., 2013).

Imbibition of the mature seed marks the end of the dry quiescent state. The seed swells, metabolic activity resumes and provided the seed is after-ripened (AR) it will proceed to germination, which in *Arabidopsis* consists of two visible events (Holdsworth et al., 2008a; Weitbrecht et al., 2011). First, the testa splits (testa rupture) due to underlying expansion of the endosperm and embryo, followed by the radicle (embryonic root) protruding through the endosperm (endosperm rupture), completing germination; in dormant (D) seeds neither testa nor endosperm rupture occur. In *Arabidopsis* seed dormancy is coat-enhanced, with both the testa and endosperm playing a critical role in dormancy maintenance (Debeaujon et al., 2000; Muller et al., 2006; Nonogaki, 2006; Bethke et al., 2007; Lee et al., 2010; Chen et al., 2014) as seen by the fact that their removal results in growth of the embryo and normal seedling establishment. Gene expression analysis of the endosperm has revealed the transcriptional dynamics and functions of this tissue (Penfield et al., 2006; Endo et al., 2012; Dekkers et al., 2013; Yan et al., 2014; Scheler et al., 2015) and, for example, revealed a role for ethylene in endosperm weakening (Linkies et al., 2009; Martinez-Andujar et al., 2012b). Seed dormancy is very dynamic and is influenced by a combination of genetic factors, environmental conditions experienced by the mother plant, as well as environmental signals perceived in the imbibed state (Auge et al., 2015; Huang et al., 2015). Furthermore, AR seeds can induce secondary dormancy (dormancy cycling) when circumstances do not permit germination (Cadman et al., 2006; Finch-Savage et al., 2007; Footitt et al., 2011), an important mechanism of persistence of seeds in the soil seed bank (Baskin and Baskin, 2014; Long et al., 2015).

Gene expression analyses on dormant and non-dormant whole seeds have been performed previously (Cadman et al., 2006; Finch-Savage et al., 2007; Carrera et al., 2008; Holdsworth et al., 2008b; Barrero et al., 2009; Bassel et al., 2011; Rajjou et al., 2012; Foley et al., 2013), such studies proved instrumental to our understanding of dormancy, dormancy release and germination. In this work we used genome-wide gene expression analysis to study the early responses upon rehydration of both D and AR seeds, with the aim to obtain a global overview of the transcriptome dynamics in spatial and temporal detail and to provide a data set that can be queried and mined by the seed community which is facilitated by the web tool we made available. For this study we followed a similar strategy as in previous studies on germinating seeds (Dekkers et al., 2013; Scheler et al., 2015) by investigating the dynamics of transcript abundance changes at four time points and in two seed compartments. Subsequently, we have used our data set to address three questions: (1) How fast do gene expression patterns differentiate between D and AR seeds after rehydration? (2) Which gene expression profiles underlie the differential gene expression between D and AR seeds? (3) Are there genes which are specifically expressed between the two dormancy states and the two seed compartments?

## Materials and Methods

### Plant Material, Growth Conditions, and RNA Extraction

*Arabidopsis* (accession of the Cape Verde Islands, Cvi, N8580) plants were grown in climate cell consisting of 16 h light/8 dark at a temperature of 22°C. After harvest seeds were stored in paper bags under ambient temperature and humidity. Dormancy release was followed by germination assays throughout the after-ripening period, and after 16 months dormancy was released. The imbibed seeds were dissected and the micropylar and chalazal endosperm (MCE) and the radicle and hypocotyl (RAD) were collected for RNA isolation. RNA of the RAD samples was isolated with the Absolutely RNA Nanoprep kit (Agilent Technologies) as we used earlier for our Col-0 seed samples (Dekkers et al., 2013). This method did not work well for Cvi MCE and dry seed samples, so RNA of the MCE and dry seed samples was isolated by an alternative method based on the hot borate isolation protocol (Wan and Wilkins, 1994), and additionally cleaned up with NucleoSpin RNA Clean-Up XS (Macherey-Nagel).

Germination assays were performed to assess dormancy release using the Germinator set-up (Joosen et al., 2010). Samples of approximately 50–100 seeds were sown on two layers of blue germination papers with 50 mL of demineralized water in plastic trays (15 cm × 21 cm). Trays were piled and wrapped in a closed and transparent plastic bag. The bags were incubated in an incubator at 22°C and continuous light. Germination was followed by taking photos daily for up to 10 days.

### Microarray Analysis

**FIGURE 1:**
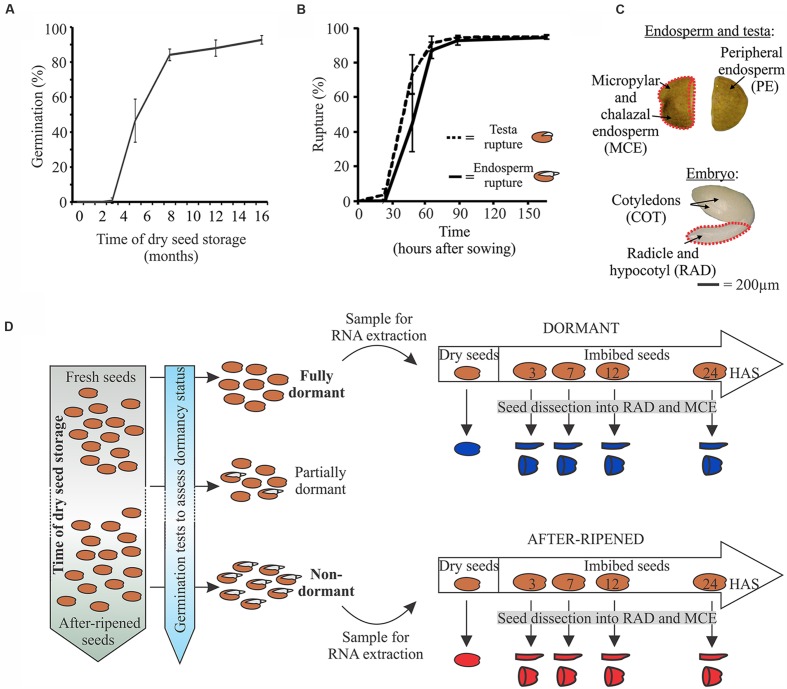
**Dormancy release of Cvi seeds and the experimental set-up for the transcriptome analysis in D and AR seeds.** **(A)** Loss of dormancy by dry seed storage (after-ripening) of the Cvi batch. Line graph shows germination percentage (average ± SD of four replicates). **(B)** Graph shows the rate of testa and endosperm rupture (average ± SD of four replicates) of seeds after 16 months of dry seed storage. **(C)** Photographs show the seed compartments used for gene expression analysis, micropylar and chalazal endosperm (MCE) and radicle+hypocotyl (RAD) indicated by the red dashed outer lines. **(D)** Picture summarizes the experimental set-up of the seed sampling. Freshly harvested seeds were fully dormant. These D seeds were imbibed for 3, 7, 12, and 24 h. The imbibed seeds were dissected to obtain two compartments, the radicle+hypocotyl (RAD) and the MCE at each time point. Together with non-dissected D dry seeds these total nine dormant samples which are indicated in blue. Next we followed AR of the seed batch by performing germination assays during the AR period. When seeds were fully capable to germinate the seeds are fully AR and thus non-dormant. Similar sampling was performed to obtain another nine AR samples, indicated in red.

This temporal and spatial sampling resulted in nine dormant and nine after-ripened samples (**Figure 1D**) and these 18 samples, with three replicates for each sample, were hybridized to Affymetrix ATH1 gene chips. These 54 chips were normalized together with the 34 chips of near isogenic lines of several *DELAY OF GERMINATION* (*DOG*) loci [including samples of D and AR seeds of L*er*-0 and near isogenic lines NIL*DOG1*, NIL*DOG2*, NIL*DOG3*, and NIL*DOG6* (Bentsink et al., 2010) that were imbibed for 24 h] and the previous 116 chips from Dekkers et al. (2013) using Robust Microarray Averaging (RMA; Irizarry et al., 2003), using a custom chip definition file (.cdf) from the CustomCDF project (Dai et al., 2005; Ath1121501_At_TAIRG.cdf v18.0.0, released 23rd January 2014, from http://brainarray.mbni.med.umich.edu/Brainarray/Database/CustomCDF), after removal of the control probes, 21315 genes remain. The microarray data described in this article has been deposited in the National Center for Biotechnology Information’s Gene Expression Omnibus (accession number GSE76907). The quality controls (**Supplementary Figure S5**; **Supplementary Table S1**) indicate that this is a robust dataset that allows us to investigate, in seeds with opposing dormancy states, the transcriptional dynamics in two key compartments that regulate seed dormancy and germination. A web tool enabling the querying of this dataset^1^, under ‘Dormancy Gene Expression Plots.’

### Differential Expression and Overrepresentation Analysis

To calculate genes as being differentially expressed at a certain fold level, the difference in means must be at least that large, and they must be statistically significant on a *t*-test at a *p*-value of 0.05. In order to reduce bias from genes whose expression is within the noise region, before fold changes are calculated the data means are first clipped at an expression level of 4 (log_2_)- replacing anything less than four with four. This may slightly underestimate the number of differentially expressed genes (or their level of fold change), but helps prevent the noise region from heavily influencing the results of the fold changes; this thresholding is reflected in the numbers in Data File S1, and all numbers throughout. The gene lists were analyzed using Genetrial (Keller et al., 2008)^2^ for overrepresented GO categories.

## Results

### Temporal and Spatial Gene Expression in D and AR Seeds

To better understand the early transcriptome changes upon sowing in D and AR seeds, we studied their temporal and spatial gene expression profiles. For this experiment we made use of the highly dormant *Arabidopsis* Cvi accession which has been frequently used in seed dormancy research (Alonso-Blanco et al., 2003; Ali-Rachedi et al., 2004; Cadman et al., 2006; Finch-Savage et al., 2007). We followed the loss of dormancy by germination assays during AR, and after 16 months of dry storage over 90% of the seeds germinated, and were therefore considered to have lost dormancy (**Figures 1A,B**). Imbibed seeds were dissected into two distinct compartments, the micropylar and chalazal end of the endosperm (MCE), which acts as a barrier together with the seed coat, and the combined radicle and hypocotyl (RAD; **Figure 1C**), which has to expand to break through the covering layers to complete seed germination. To capture the transcriptome dynamics, we conducted microarray analysis on RNA isolated from dry seeds and the two compartments at four time points, i.e., 3, 7, 12, and 24 hours after sowing (HAS), of both D and AR seeds (**Figures 1C,D**). These compartments and time points were chosen to align with the sampling of germinating Col-0 seeds we performed previously (Dekkers et al., 2013). Three HAS was chosen as the earliest time point since at this stage initial transcriptional changes can be clearly observed (Preston et al., 2009; Dekkers et al., 2013; Scheler et al., 2015).

**FIGURE 2:**
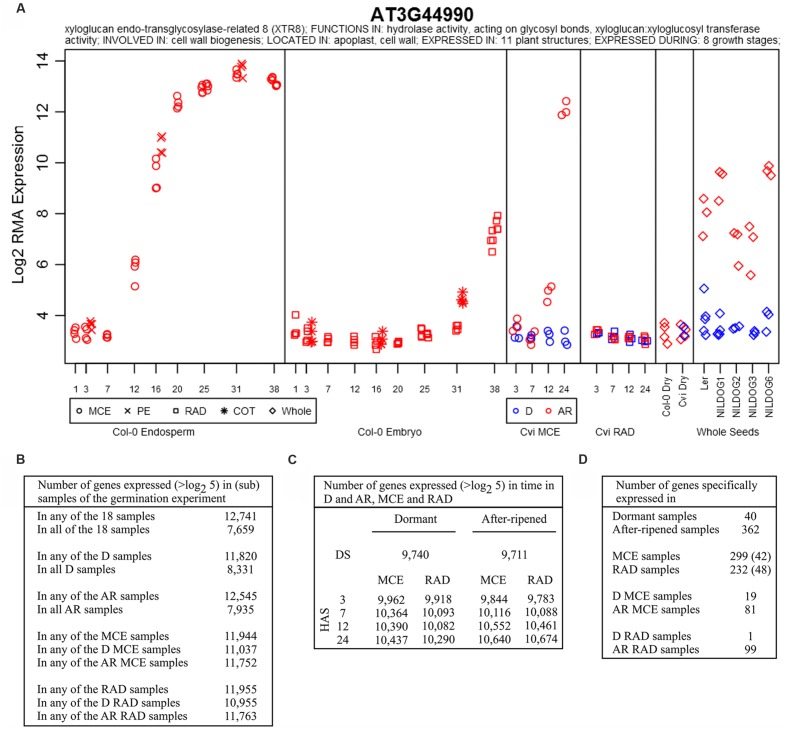
**Spatial and temporal gene expression in the Cvi seed dataset.** **(A)** Gene expression plot from the web tool showing the expression of *XTR8* as an example in various seed samples. **(B)** Number of expressed genes in various (sub) samples. **(C)** Number of genes expressed at different time points after sowing. **(D)** Numbers of specifically expressed genes in seed compartments, dormancy state or combined. The seed compartment specific sets were further refined using the gene expression data set of dissected germinating Col-0 seeds, with. the 42 MCE specific genes not present (expression value < 5 log_2_) in Col-0 RAD, cotyledons and peripheral endosperm as well and similarly, the 48 genes specific to the RAD are not present in the Col-0 cotyledons and endosperm samples. D, dormant; AR, after-ripened; MCE, micropylar and chalazal endosperm; RAD, radicle and hypocotyl; HAS, hours after sowing.

A freely available web tool^3^ (under ‘Dormancy Gene Expression Plots’) was developed in which the gene expression data of these dormancy samples were combined with the earlier mentioned seed germination samples (Dekkers et al., 2013). The tool also includes transcriptome data of near isogenic lines of *DELAY OF GERMINATION* (NIL*DOG*) loci, providing gene expression information concerning D and AR seeds of several genotypes with different primary dormancy levels (Leónie Bentsink, personal communication). The expression profile of a gene of interest can be visualized across all these samples, as was previously done for anther and flower microarray datasets (Pearce et al., 2015). The resulting plots show how genes are expressed across these three experiments (**Figure 2A**), and provides a useful tool for the community, allowing quick determination of the behavior of a gene of interest.

### Analysis of the Number of Differentially Expressed Genes in Different Dormancy States and Seed Compartments

Over the whole time course 12,741 genes were detected to be expressed (>log_2_ 5, 59.8% of the 21,315 genes on the chip) in at least one of the 18 samples, while 7,659 genes (60.1% of all expressed genes) were expressed in all 18 samples (**Figure 2B**). In dry seeds and at the early time points fewer genes were expressed, this number increased during the time course in the two compartments (MCE and RAD) and in both dormancy states (**Figure 2C**). We detected nearly equal numbers of genes expressed in the MCE and the RAD (11,944 vs. 11,955 respectively) over the time course, but fewer genes were expressed in the D samples (11,820) compared to the AR samples (12,545).

We identified transcript sets that were specifically expressed in either the D or the AR state, by considering genes which were expressed in one state above 6 (on a log_2_ scale) and expressed below 5 (below which expression becomes indistinguishable from technical noise on the microarray) in the other state. According to this definition, 9,735 (88.5% of the 11,002 genes expressed over 6) of the genes are shared between both states whilst 40 (0.4%) are specific to D and 362 (3.3%) are specific to AR seeds. The remaining 865 transcripts (7.9%) are expressed over 6 in one state but between 5 and 6 in the other state, and are not classed as being highly specific to either physiological state. Similarly, a number of transcripts were specific to the MCE (299 genes) and the RAD (232 genes) which could be further refined (to 42 genes for the MCE and 48 for the RAD) using the transcript information of the Col-0 seeds (Dekkers et al., 2013, **Figure 2D** and Data File S1). Subsequently, we investigated genes that were both state (D or AR) and compartment (MCE or RAD) specific, although, these resulted in very small gene sets. We identified 19 transcripts specific for the D MCE while in the D RAD only one such gene was found. Higher numbers of specifically expressed genes were found in the AR state (81 in the MCE and 99 in the RAD, **Figure 2D**).

### Dormant Seeds Show an Initial, Transient Phase of Differential Gene Expression after Rehydration

**FIGURE 3:**
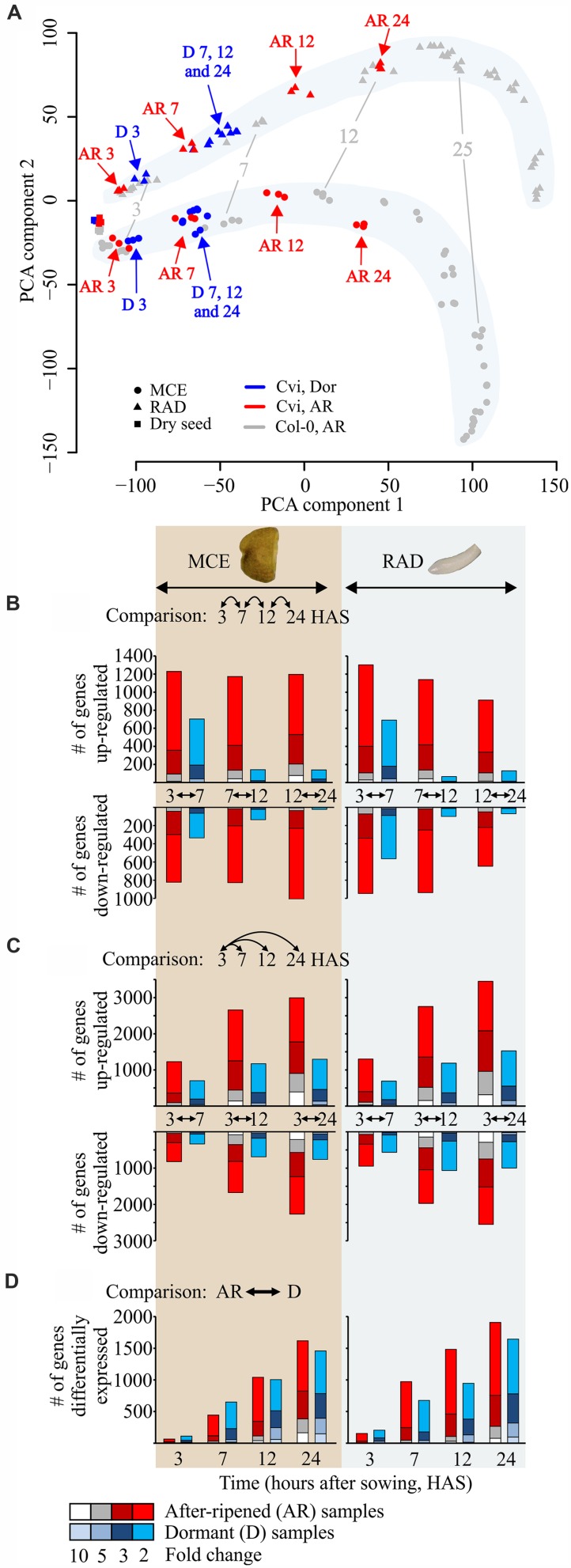
**Transcriptional dynamics in D and AR Cvi seeds.** **(A)** Principal component analysis plot of D (in blue) and AR (in red) Cvi (samples, combined with the AR Col-0 samples (in gray, from Dekkers et al., 2013). Dry seed samples are depicted by squares, MCE samples by circles and RAD samples by triangles. Numbers indicate the hours after sowing, and individual replicates are plotted. **(B)** Number of differentially expressed genes between consecutive time points. **(C)** Number of differentially expressed genes compared to the first time point (3 HAS). **(D)** Number of differentially expressed genes with higher expression in either D (blue) or AR (red/gray/white) seed compartments by comparing D and AR samples at each time point (e.g., D vs. AR MCE 3 HAS), colors in each bar indicate the number of differentially expressed at 2, 3, 5, and 10 fold change cut-offs.

We performed a principal component analysis (PCA) to globally investigate the transcriptome time course. In our previous study of AR Col-0 seeds (Dekkers et al., 2013), the PCA analysis separated the samples mainly by time and tissue type. To be able to compare both studies we plotted the D and AR Cvi samples together with these AR Col-0 samples (**Figure 3A**). Like the AR Col-0 samples, the AR Cvi samples are mainly separated along the first principal component (PC), with that PC appearing to represent time. The AR Cvi samples do ‘move’ slower though, and in particular the later time points (12 and 24 HAS) lag behind those of the AR Col-0, which most likely reflects the slower germination rate compared to the AR Col-0 seeds (germination speed calculated as the time required to reach 50% of germination (T_50_) was 49 h for Cvi compared to 39 h for Col-0). Intriguingly, the D samples show a very different behavior; although the D samples cluster with the AR samples at 3 and 7 HAS, at later time points (12 and 24 HAS) the samples ‘stall’ around the 7 HAS position in the PCA plot (**Figure 3A**). This indicates that limited transcriptional changes are occurring at the later time points in the D samples, and is supported by the number of differentially expressed genes detected between the consecutive time points in both compartments (**Figure 3B**). The number of differentially expressed genes (over threefold up and down) between 3–7, 7–12, and 12–24 HAS in the AR MCE/RAD were 656/740, 614/667 and 760/558 genes, respectively. For the D samples these numbers are considerably lower, most notably between the later time points, i.e., 259/270, 45/27, and 40/30 (**Figure 3B**). Over the whole time course (3–24 HAS), the total number of differentially expressed genes in the AR state is over 3000 in both tissues (MCE 3018, RAD 3601), which is over four times more than in the D samples (MCE 682, RAD 821; **Figure 3C** and Data File S1).

### Transcriptome Dynamics in the Endosperm of D and AR Seeds

**FIGURE 4:**
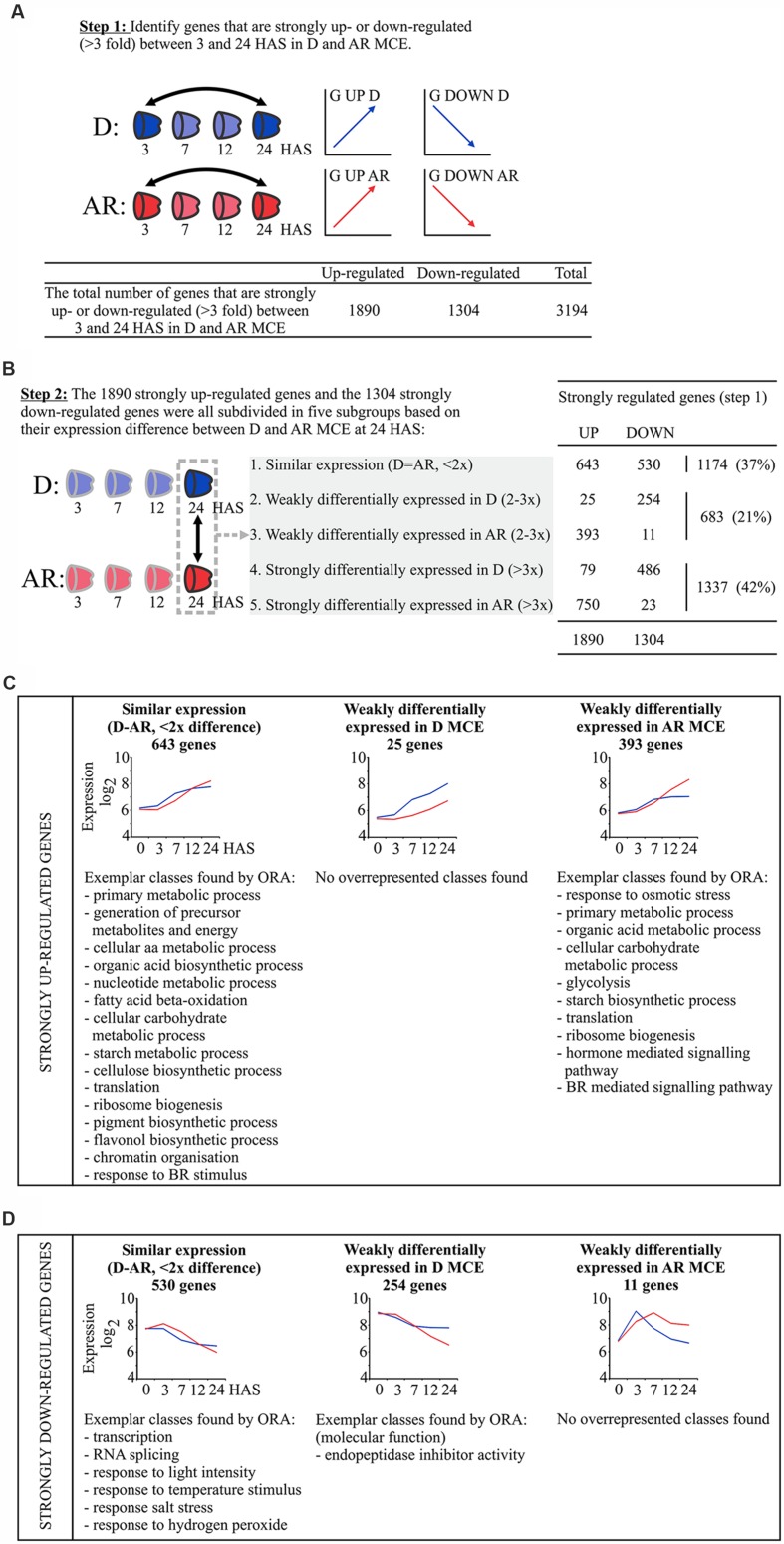
**Analysis of temporally regulated genes in D and AR MCE using a two-step approach.** **(A)** Step 1: filter for genes which are at least threefold changed between 3 and 24 HAS, total numbers are indicated underneath. **(B)** Step 2: divide temporally changed genes into five classes based on their expression between D and AR samples at 24 HAS. **(C)** Depicts the 1890 temporally up-regulated genes and **(D)** the 1304 temporally down-regulated genes those that get similarly expressed or weakly differentially expressed between D and AR MCE at the last time point (24 HAS; step 2). For these classes, expression profiles (obtained by averaging over all genes in each set) and exemplar GO classes found by overrepresentation analysis are shown. The blue (red) lines indicate the averaged expression in the D (AR) samples. Genes that become strongly differentially expressed in the D or AR MCE are depicted in **Figure 5**.

To get a more detailed view of the transcriptome similarities as well as the differences (and the gene expression profiles underlying these) in D and AR seeds we employed a two-step approach using the MCE samples. Since there is differential gene expression between D and AR seeds during the time course of imbibition, we have first focused on transcripts with a strong temporal regulation (which are identified in the first step, **Figure 4A**). We identified 3194 genes which show a strong difference in expression (defined as threefold change) during the germination time course by comparing 3 and 24 HAS in D and/or AR MCE, of which a total of 1890 genes were strongly up-regulated, whereas 1304 genes were strongly down-regulated (**Figure 4A**). In the second step, we tested whether these strongly differentially regulated genes are differentially expressed between D and AR MCE at the last time point (i.e., the comparison D MCE 24 HAS vs. AR MCE 24 HAS; **Figure 4B**). We focused on genes that are similarly expressed (an expression difference less than twofold) or differentially expressed (threefold difference) in either D or AR state. A smaller set was identified that contained genes that were neither similarly expressed nor strongly differentially expressed (showing a difference in expression between 2- and 3-fold) and these are denoted as weakly differentially expressed. Since we have selected for strongly temporally regulated genes during germination (step 1, **Figure 4A**), this suggests that these gene expression profiles are similar, although less pronounced in one or the other state (**Figures 4C,D**). Based on this analysis these temporally regulated genes were subdivided in five classes: (1) genes that are similarly expressed between D and AR MCE, (2) genes that are weakly differentially expressed in the D MCE, (3) genes that are weakly differentially expressed in the AR MCE, (4) genes that are strongly differentially expressed in D MCE, and (5) genes that are strongly differentially expressed in AR MCE (**Figure 4B**).

### Temporally Regulated Genes that are Similarly Expressed in the D and AR Endosperm

We first investigated genes that are eventually expressed at a similar level (i.e., an expression difference of less than twofold) in D and AR MCE at 24 HAS. These represent transcriptional changes that occur in response to rehydration, independent from the physiological state (D or AR). In total we found 3194 genes that are strongly regulated between 3 and 24 HAS in the MCE (**Figure 4A**). Of this set 1174 genes (37%, 643 genes up and 531 genes down) become eventually similarly expressed (**Figure 4B** and Data File S1). In **Figures 4C,D**, the expression profiles of the categories identified in the second step are indicated. From the germination up-regulated gene set (643 genes), overrepresentation analysis (ORA) of gene ontology (GO) classes showed the presence of genes related to key metabolic processes related to amino acid, organic acid, nucleotide and carbohydrate as well as chromatin organization and translation, among others. The 531 down-regulated genes have functional classes related to transcription, RNA processing and stress overrepresented.

### Temporally Regulated Genes that are Strongly Differentially Expressed in the D or AR Endosperm

**FIGURE 5:**
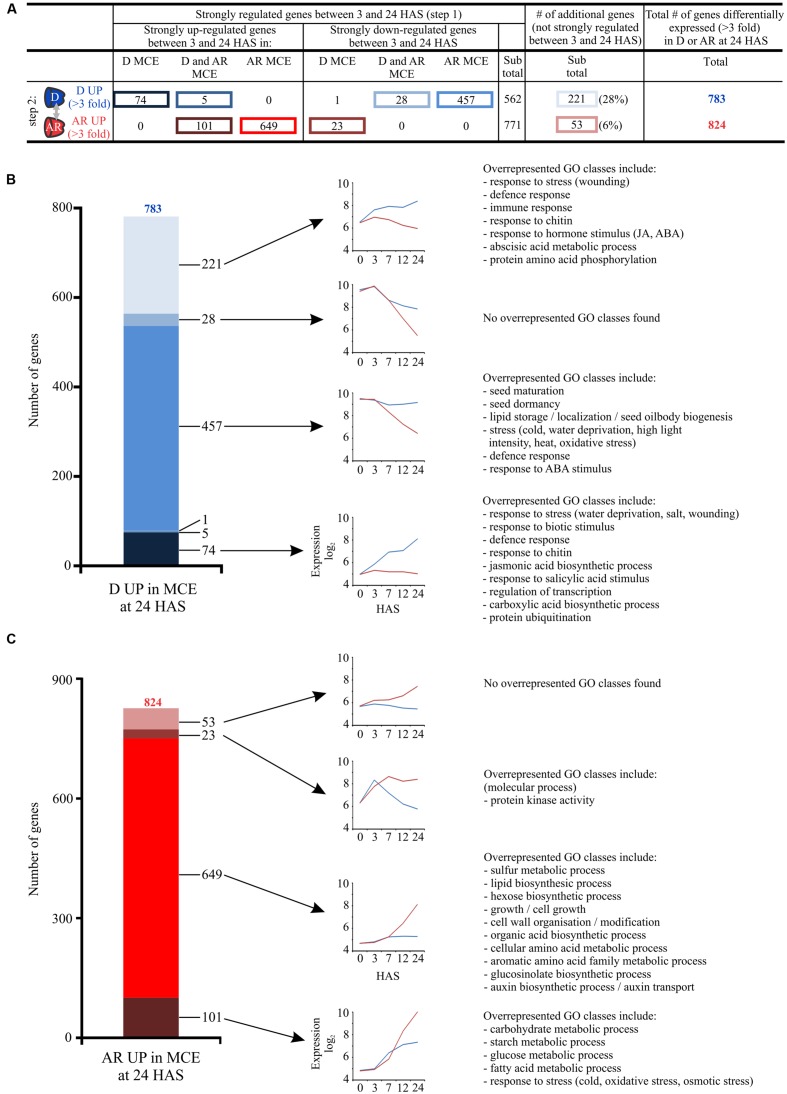
**Analysis of gene expression profiles that contribute to differential gene expression between D and AR MCE at 24 HAS.** **(A)** In total 783 genes are higher expressed in the D MCE and 824 genes higher in the AR MCE at 24 HAS, using a threefold cut-off. The table shows the expression profiles (between 3 and 24 HAS in D and AR MCE that contribute to the differential expression between D and AR MCE at 24 HAS. The strongly up- and down-regulated genes (step 1) are further subdivided in those that are strongly regulated in the D MCE, D and AR MCE and AR MCE between 3 and 24 HAS. **(B)** The graph depicts the 783 genes of the D set (D up-regulated vs. AR at 24 HAS in the MCE). Of the four largest groups the expression profiles and exemplar GO classes found by overrepresentation analysis are shown. **(C)** The graph depicts the 824 genes of the AR set (AR up-regulated vs. D at 24 HAS in the MCE). Of the different groups expression profiles and exemplar GO classes found by overrepresentation analysis are shown. The expression profiles were obtained by averaging the log_2_ expression values of all genes in each particular set over all time points where time point 0 means the dry seed stage. The blue (red) lines indicate the averaged expression in the D (AR) samples. The selection presented in **(A)** is not mutually exclusive, five genes are both strongly up-regulated in one state and strongly down-regulated in the other and were therefore counted twice, leading to the row subtotals deviating slightly from the total.

A significant fraction (1337 out of 3194 genes, 42%) of temporally regulated genes become strongly differentially expressed in either D (564 genes) or AR (773) MCE at 24 HAS (**Figure 4B**). We subdivided the temporally strongly up- and down-regulated genes (from step 1) into those that are strongly up-regulated in the D MCE, D and AR MCE and AR MCE between 3 and 24 HAS and similar subdivision was made for the down-regulated genes (see **Figure 5A**). Furthermore, these sets were then subdivided based on whether the genes are strongly differentially expressed in the D or AR endosperm (step 2, **Figure 5A**). These however, do not represent the complete list of genes which are differentially expressed between the D and AR states in the MCE at 24 HAS (as they contain 783 differentially expressed genes in D and 824 differentially expressed genes in AR using a threefold cut-off, **Figure 3D**, Data File S1). This is explained by the presence of genes that are not strongly regulated during germination, which instead have opposing gene expression profiles between D and AR between 3 and 24 HAS, and these are also included in **Figure 5**.

A total of 783 genes are strongly differentially expressed in D MCE (vs. AR MCE at 24 HAS, **Figure 5A**). Four major gene expression profiles underlie this differential gene expression (containing at least 20 genes). The largest gene expression profile (457 genes, 58%) contains genes that are actually down-regulated in AR MCE (**Figures 5A,B**). ORA of this group identified GO functional classes related to germination/dormancy, lipid storage and stress (**Figure 5B**). The second largest profile in this D set consists of 221 genes (28% of 783 genes) that are not strongly temporally regulated after imbibition (less than threefold change between 3 and 24 HAS). The strong differential expression of the D set is explained by opposing gene expression profiles between D and AR when comparing 3 and 24 HAS (**Figure 5B**), with ORA showing the accumulation of stress and hormone related gene classes in D MCE. Less than 10% of the D UP genes are strongly up-regulated during germination in D but not in AR MCE (a set of 74 genes) which is overrepresented for genes whose function is related to response to abiotic and biotic stresses.

Four gene expression profiles underlie the strong activation of 824 genes in the AR MCE (**Figures 5A,C**). The largest group (649 genes, 79%) is strongly up-regulated during the time course in AR MCE, but not in D MCE (**Figures 5A,C**); these genes encode functions related to primary and secondary metabolism, growth and cell wall modification and are germination related. The second largest group consists of 101 genes that are strongly up-regulated (threefold) in both D and AR but are even stronger up-regulated in the AR MCE. This gene expression profile resulted in genes differentially expressed in AR MCE whose functions are related to carbohydrate and fatty acid metabolism and stress response. Interestingly, these classes overlap with those identified among the genes which became similarly expressed in AR and D MCE and those that became weakly differentially expressed in the AR MCE (see **Figure 4C**). Overall these analyses reveal multiple gene expression profiles that are responsible for the differential gene expression between D and AR endosperm, with ORA showing that different patterns regulate the expression of genes related to various processes.

### MCE vs. RAD: Genes Related to Biotic and Abiotic Stress are Differentially Expressed in the D MCE

The transcriptional dynamics in D and AR RAD, using this two-step approach, showed similar trends (**Supplementary Figure S1**). In the RAD a total of 3781 genes were found to be strongly regulated (up and down) between 3 and 24 HAS (step 1), of which 39% became eventually similarly expressed in both D and AR RAD, 26% became weakly and 35% became strongly differentially expressed in either the D or AR RAD (see **Supplementary Figure S1** for details). The transcriptional differences (as well as the gene expression profiles underlying these) in D and AR RAD are shown in **Supplementary Figure S2**. At 24 HAS 780 genes were detected to be higher expressed in the D RAD, with the majority of them being down-regulated over our time course in the AR RAD although not in the D RAD (581 genes, 74%). This is similar compared to the D MCE set, although in the MCE its relative contribution is somewhat smaller (58%, see above) which is explained by a relatively larger fraction of genes with opposing gene expression profiles between the D and AR state (compare **Figure 5** and **Supplementary Figure S2**). We detected a total of 755 genes up-regulated in the AR RAD at 24 HAS, of which the largest group (575 genes, 76%) was strongly up-regulated during our time course in AR RAD but not in the D RAD.

**FIGURE 6:**
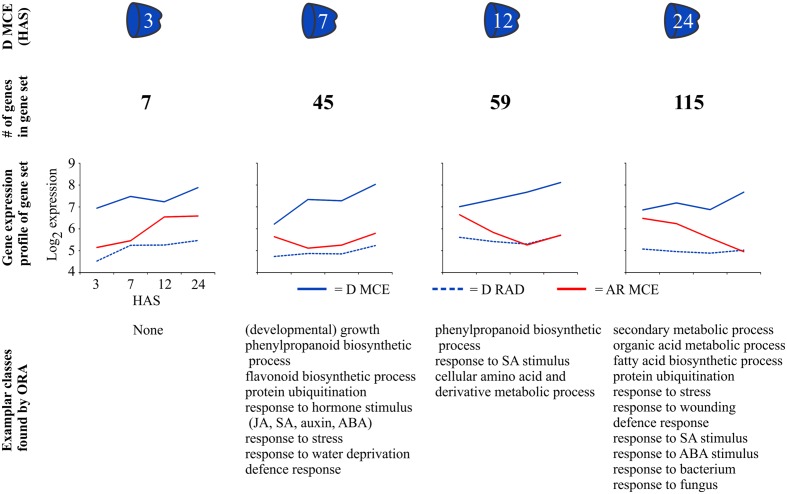
**Identification and analysis of gene sets that are differentially expressed in the D MCE.** We used fold change differences to obtain gene sets that are enhanced in the D MCE at each individual time point. The genes selected are at least threefold higher expressed in the D compared to the AR state and threefold higher expressed in the MCE compared to the RAD. The gene sets identified in the D MCE at 3, 7, 12 and 24 HAS, ranged from 7 to 115 genes in size. Underneath the mean log_2_ expression of each gene set is plotted in the D MCE (blue solid line), D RAD (blue dashed line) and the AR MCE (red solid line). Under the graphs exemplar GO classes, found using ORA analysis, are indicated.

Finally, we employed a strategy using fold change differences to obtain gene sets that are enhanced in either D MCE or D RAD at each individual time point to investigate whether such gene sets reveal functional differences between the both compartments in the D state. The genes selected are at least threefold higher expressed in the D state and threefold higher expressed in either the MCE or the RAD. Using this strategy, we identified gene sets that are differentially expressed in D MCE (**Figure 6**) and D RAD (**Supplementary Figure S3**). The gene sets identified in the D MCE contained 7, 45, 59, and 115 genes at the time points 3, 7, 12, and 24 HAS, respectively. Using ORA of these sets identified functional classes related to abiotic and biotic stresses, protein ubiquitination and secondary metabolism (**Figure 6**). In comparison to the D MCE, relatively small gene sets (ranging between 11 and 30 genes) were identified in the D RAD and only a single overrepresented gene class was found (**Supplementary Figure S3**).

## Discussion

Seed dormancy is an important trait both in natural and farmed ecology, and issues related to seed dormancy have implications for human nutrition (Bewley et al., 2013). Either too little or too much dormancy can cause reduced crop yield and substantial economic losses (Gubler et al., 2005; Dekkers and Bentsink, 2015), so insights into this process are important as they can potentially be translated into practical applications (Gerjets et al., 2010; Martinez-Andujar et al., 2012a; Nambara and Nonogaki, 2012). Here we report a microarray analysis of D and AR seeds over the first 24 HAS of two seed compartments that are important in the regulation of seed dormancy and germination (Nonogaki, 2014; Bassel, 2016). Previous gene expression studies comparing D and AR seeds have been used to identify seed dormancy regulators (Barrero et al., 2010) and this set can be similarly used for data mining, particularly through use of the webtool, which has been developed to aid data visualization and exploration. Moreover, the dataset allows us to answer several specific questions, e.g., concerning the profiles underlying differential expression in D and AR seeds, how fast gene expression changes after rehydration and whether genes exist that are specifically expressed between the two dormancy states and two seed compartments which will be addressed below.

### The Larger Differential Gene Expression Activity of AR Seeds as Compared to D Seeds is the Main Driver for Differential Gene Expression between D and AR Seeds

In our study we identified genes that are strongly differentially expressed (up or down) in both dormancy states in the time course between 3 and 24 HAS in the MCE (3194 genes found) and RAD (3781 genes found). Next, we investigated whether these genes were similarly or differentially expressed between D or AR MCE and RAD at 24 HAS. This revealed that a large proportion of those (37–39%) are expressed in a similar manner in D and AR seed compartments. Seed germination involves re-activation of, and changes in, cellular activity (Fait et al., 2006; Weitbrecht et al., 2011; Rosental et al., 2014), with metabolism resuming in both imbibed dormant and non-dormant seeds (Bewley, 1997). The gene sets that are strongly enhanced in both D and AR seeds (MCE and RAD) are overrepresented for GO classes including key cellular metabolic processes like translation, amino acid, organic acid, nucleotide and carbohydrate metabolism (**Figure 4**; **Supplementary Figure S1**). A total of over 500 genes were similarly down-regulated in expression in D and AR seed compartments. Overrepresented GO categories in both MCE and RAD included transcription and response to stress and other environmental cues. These genes likely represent remnants of the dry state or seed maturation that are repressed in rehydrated seeds regardless of their dormancy status.

The remaining genes become differentially expressed (**Figure 4B**; **Supplementary Figure S1**). Noteworthy is the finding that an overwhelming majority of the temporally down-regulated transcripts become differentially expressed in the D MCE whereas a vast majority of the temporally up-regulated genes become differentially expressed in the AR MCE with similar observations made in the RAD (**Figure 4**; **Supplementary Figure S1**). Among the genes that are higher expressed in AR MCE and RAD the GO classes primary metabolic process, cellular carbohydrate metabolic process, translation, and ribosome biogenesis were overrepresented (**Figure 4C**). This group of genes is activated in both D and AR seeds, but with a higher relative abundance in the AR state, likely to support the higher cellular activity and metabolic demand to support seed germination and subsequent seedling growth.

Using this transcriptome data, we set out to address three questions. The first question we aimed to answer is which profiles underlie the differential gene expression between D and AR seeds, and we described these patterns, genes and the functional classes they represent (**Figure 5**; **Supplementary Figure S2**; Supplemental Data File S1). Although we identified four major gene expression profiles for each, they show striking differences. Most genes that become higher expressed in D seeds are actually down-regulated in AR seeds (both MCE and RAD). As this down-regulation does not occur (or not to the same extent) in the D seeds, these transcripts accumulate eventually at a higher relative abundance in the D samples. This set includes genes related to seed maturation, seed dormancy, lipid storage and response to stress. Only a small proportion of the genes which are up-regulated during the time course in D seeds fail to do so in AR seeds (74 genes in the MCE, 38 in the RAD) and both sets contain genes related to (a)biotic stress resistance. In contrast, the largest number of genes that become higher expressed in AR seeds are strongly up-regulated during the time course in AR seeds, but not (or to a lesser extent) in D seeds. Genes in this set include those involved in (cell) growth and cell wall modification, among others that are important for seed germination. In conclusion, considering all genes that become, either weakly or strongly, differentially expressed within the dormancy states, with the overwhelming majority of temporally down-regulated transcripts becoming higher in the D MCE (740 out of 774, 95.6%) and the vice versa in the AR MCE (1143 out of 1247, 91.7%; **Figure 4B**), with similar proportions in the RAD. This high directional overlap emphasizes how the genes which are changing in time are changing more in the AR seeds than the D seeds.

Indeed, transcriptome changes within D seed compartments were mainly observed in a transient fashion early after sowing, with a dramatic drop thereafter resulting in a clearly reduced number of changes over the first 24 HAS in D compared to AR seed compartments. However, the genes that increase or decrease in relative abundance over the first 24 HAS in D samples are not very different since these largely overlap with genes differentially expressed in AR samples (**Supplementary Figure S4**). Thus, although the level of differential gene expression is far less in D samples, the genes that do change upon rehydration are largely similar to those that change in AR samples. In conclusion, it is this huge difference in the number of transcriptional changes (both up and down) in the AR seeds in contrast to D seeds, which mainly drives differential gene expression between D and AR samples.

### Differential Gene Expression is Rapidly Detected between Imbibed Dormant and after-Ripened Seeds

After-ripening overcomes seed dormancy and allows germination in appropriate conditions (Bewley, 1997; Finch-Savage and Leubner-Metzger, 2006; Holdsworth et al., 2008a). Although linked to dormancy release, the after-ripening response was also observed in stored non-dormant genotypes (Carrera et al., 2008). After-ripening induces changes in seeds during dry storage; the mechanism by which this occurs is unclear, although it may include reactive oxygen species (ROS; Oracz et al., 2007; Bewley et al., 2013; El-Maarouf-Bouteau et al., 2013). We used dry seed after-ripening for 16 months to break dormancy of the D Cvi seeds. In our experiment the transcriptome differences between D and AR dry Cvi seeds are low (only seven genes show a twofold difference), indicating that genome-wide reprogramming of transcript abundances occurs mainly after water uptake (Carrera et al., 2008). This raises the second question of how quickly seeds detect and respond to the changes that occurred during dry storage. Transcriptome differences are already present at 3 HAS (which become progressively pronounced at the subsequent time points, **Figure 3D**) showing that seeds respond rapidly after rehydration to the changes that occurred during after-ripening in the dry seed suggesting that after-ripening pre-sets the transcriptional response following the initiation of imbibition.

Although the first changes between D and AR samples are visible at 3 HAS, the transcriptomes differ more strongly at 12 and 24 HAS, indicated by the different positions of the D and AR samples in the PCA plot at these time points (**Figure 3A**). It also indicates that the transcriptome of dormant seeds and that of seeds that progress toward germination start to deviate strongly between 7 and 12 HAS. Investigation of genes differentially expressed at the 7 HAS time point between D and AR samples could reveal potential transcriptional regulators that control these different transcriptional responses. Among the genes which are higher expressed (at a threefold cut-off) in both D MCE and D RAD at 7 HAS are 17 TF (of which 9 are of the ERF/AP2 class, for details see Data File S1). The gene annotations suggest that most of these TFs are related to (a)biotic stress responses indicating that the dormant seed prepares to cope with suboptimal environmental conditions. Similarly, we looked into genes which are higher expressed in the AR MCE and AR RAD at 7 HAS and identified nine TFs (Data File S1). These TFs may include candidates that are responsible for the different gene expression responses of D and AR seeds.

### The Dormant Endosperm Shows a Higher Relative Abundance of Stress-Related Genes

Lastly, we questioned whether we could identify gene sets that are both dormancy and seed compartment specifically expressed. We found very few genes (40, see **Figure 2D**) that were D specific in our data set. Many dormancy up-regulated transcripts are present in dry (and early imbibed) seeds and are not (or to a lesser degree) down-regulated in D compared to AR MCE and RAD (as discussed above). This explains why so few D specific genes were detected, which was even more obvious when we looked for genes that were both D and seed compartment specific, with only 19 and one genes found for D MCE and RAD, respectively (**Figure 2D**). Dormancy is regulated by the balance between two hormones ABA (required for dormancy induction) and GA (required for germination; Finch-Savage and Leubner-Metzger, 2006; Finkelstein et al., 2008; Bassel, 2016). Among the 19 genes in the D MCE set are a gene involved in ABA biosynthesis (*NCED2*) and another involved in GA degradation (*GA2Ox6*; Data File S1). This suggests that seed compartment specific expression of hormone metabolism genes contribute to the control of ABA/GA balance in dormant seeds.

Thus gene sets which are specific for D MCE and D RAD were identified although these were rather small. Therefore, we aimed to obtain larger gene sets which we could investigate using GO overrepresentation analysis. For this reason we employed a strategy using fold change differences to obtain gene sets that are enhanced in either D MCE or RAD at each individual time point. This resulted in larger gene sets, of which ORA identified functional classes related to abiotic and biotic stresses in the dormant endosperm (**Figure 6**). Stress related genes and proteins were previously found to be overrepresented in the endosperm of germinating *Arabidopsis* seeds and in its close relative cress (*Lepidium sativum*; Muller et al., 2010; Endo et al., 2012; Dekkers et al., 2013). Interesting in this respect, is a recent study using co-expression network analysis of *Medicago* (*Medicago truncatula*) seed maturation (Righetti et al., 2015), which revealed that biotic stress related genes were linked to seed longevity. The endosperm is the outermost living cell layer of the seed that is in contact with the environment, so the relatively higher abundance of (a)biotic stress related genes suggests a role for the endosperm in stress signaling and response. *Arabidopsis* mutants that have disturbed integrity of the testa or the cuticle layer are affected in seed germination, dormancy and longevity (Debeaujon et al., 2000; De Giorgi et al., 2015). Our results show that the expression of stress related genes is further enhanced in the endosperm of dormant compared to non-dormant seeds, the presence of which may support the survival of the dormant seed in its environment.

## Author Contributions

BD, RvB-V, and LB performed laboratory work; SP performed bioinformatic analysis and made the web tool; BD and SP analyzed data; BD, SP, and LB wrote the paper; MH and LB supervised the project; all authors contributed to the design of this study and all authors read, commented on and approved the manuscript.

## Conflict of Interest Statement

The authors declare that the research was conducted in the absence of any commercial or financial relationships that could be construed as a potential conflict of interest.

## Footnotes

1 http://www.wageningenseedlab.nl/resources

2 http://genetrail.bioinf.uni-sb.de

3 http://www.wageningenseedlab.nl/resources
