# Supplementary material for: Dormant and after-Ripened Arabidopsis thaliana Seeds are Distinguished by Early Transcriptional Differences in the Imbibed State
Source: Front Plant Sci. 2016 Aug 30;7:1323. doi: 10.3389/fpls.2016.01323 (PMC5003841; doi:10.3389/fpls.2016.01323)
Supplement: Supplementary file 1 [file Data_Sheet_1.PDF]

**Supplemental Figures S1-S5 and Table S1 with the Dekkers BJW, Pearce SP et al. paper concerning the transcriptome analysis in D and AR seeds in spatial and temporal detail.**

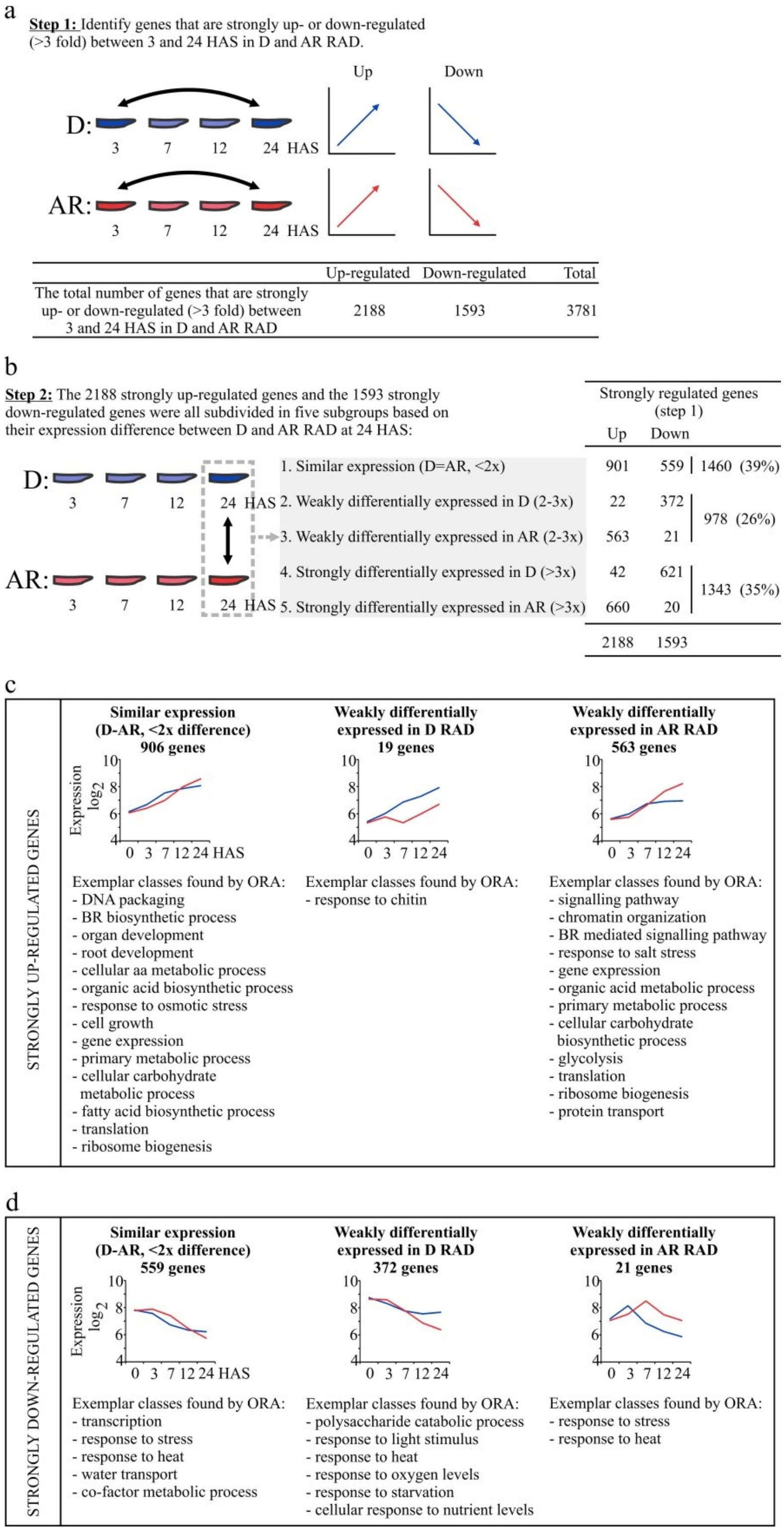


**Figure S1. Analysis of strongly regulated genes in D and AR RAD using a two-step approach.** (a) Explanation of the first step to analyse the strongly regulated genes upon rehydration. Underneath the total numbers of strongly up- or down-regulated genes detected between 3 and 24 HAS in the RAD are indicated. (b) Shows the second step. In this way we tested whether these strongly regulated genes (identified in step 1) become differentially expressed between D and AR RAD at the last time point (i.e. the comparison D RAD 24 HAS vs AR RAD 24 HAS). Based on this analysis both groups (the 2188 up-regulated and the 1593 down-regulated genes) were further subdivided in five classes. (c) Depicts the 2188 strongly up-regulated genes and (d) of the 1593 strongly down-regulated genes, those that get similarly expressed and weakly up-regulated between D and AR RAD at the last time point (24 HAS) (step 2). For these classes, expression profiles and exemplar GO classes found by overrepresentation analysis are shown. The expression profiles were obtained by averaging the $log_2$ expression values of all genes in each particular set over all time points where time point 0 means the dry seed stage. The blue (red) lines indicate the averaged expression in the D (AR) samples. Genes that become strongly differentially expressed in the D or AR MCE are depicted in Figure S2.

a

| | Strongly regulated genes between 3 and 24 HAS (step 1) | | | | | | | # of additional genes (not strongly regulated between 3 and 24 HAS) | Total # of genes differentially expressed (>3 fold) in D or AR RAD at 24 HAS |
|---|---|---|---|---|---|---|---|---|---|
| | Strongly up-regulated genes between 3 and 24 HAS in: | | | Strongly down-regulated genes between 3 and 24 HAS | | | | | |
| | D RAD | D and AR RAD | AR RAD | D RAD | D and AR RAD | AR | Sub total | Sub total | Total |
| step 2: D UP (>3 fold) | 38 | 2 | 2 | 0 | 40 | 581 | 660 | 120 (15%) | 780 |
| AR UP (>3 fold) | 0 | 85 | 575 | 18 | 2 | 0 | 676 | 79 (10%) | 755 |

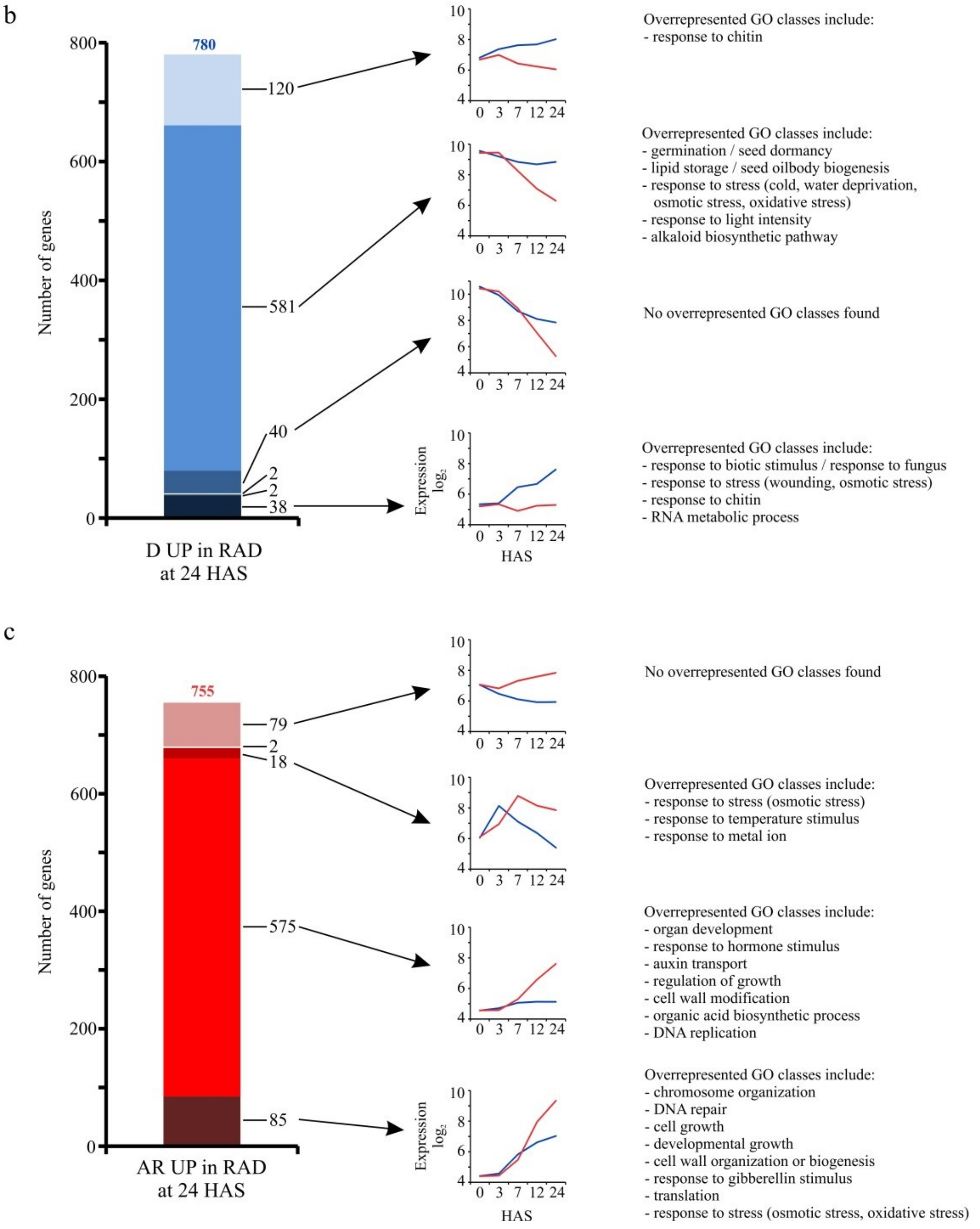


**Figure S2. Analysis of gene expression profiles that contribute to differential gene expression between D and AR RAD at 24 HAS.** (a) In total 780 genes are differentially expressed in the D RAD and 755 genes in the AR RAD at 24 HAS using a 3 fold cut-off. The table shows the expression profiles between 3 and 24 HAS in D and AR RAD that contribute to the differential expression between D and AR RAD at 24 HAS. The strongly up- and down-regulated genes (step 1) are further subdivided in those that are strongly regulated in the D RAD, D and AR RAD and AR RAD between 3 and 24 HAS. (b) The graph depicts the 780 genes of the D set (D up-regulated vs AR at 24 HAS in the RAD). Of the four largest groups the expression profiles and exemplar GO classes found by overrepresentation analysis are shown. (c) The graph depicts the 755 genes of the AR set (AR up-regulated vs D at 24 HAS in the RAD). Of the different groups expression profiles and exemplar GO classes found by overrepresentation analysis are shown. The expression profiles were obtained by averaging the $\log_2$ expression values of all genes in each particular set over all time points where time point 0 means the dry seed stage. The blue (red) lines indicate the averaged expression in the D (AR) samples. The selection presented in (a) is not mutually exclusive, seven genes are both strongly up-regulated in one state and strongly down-regulated in the other and were therefore counted twice, leading to the row subtotals deviating slightly from the total.

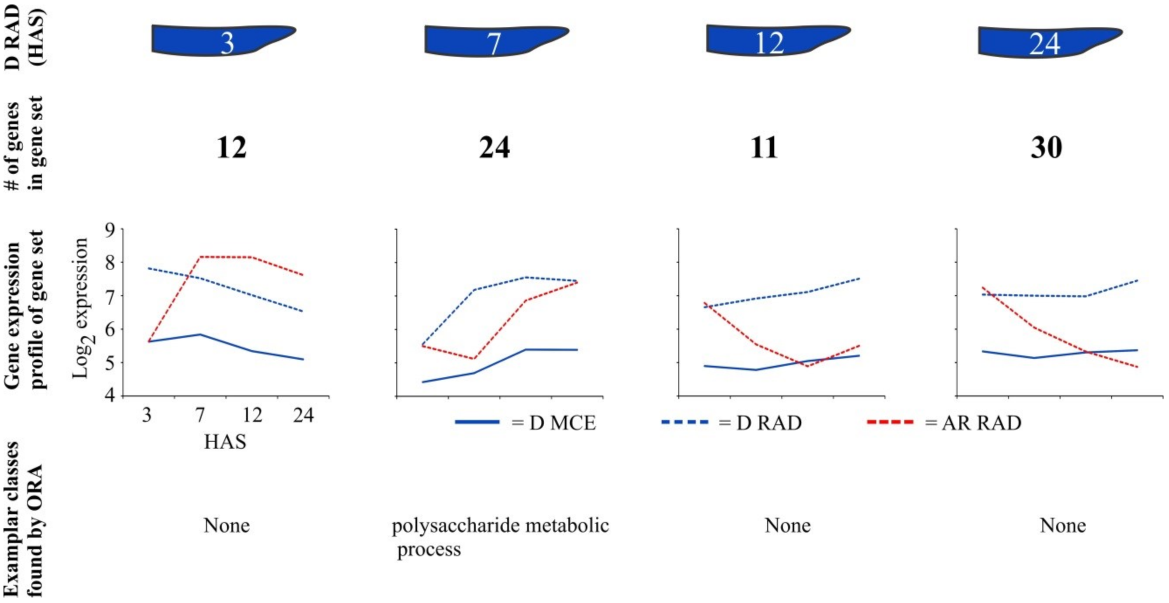


**Figure S3. Identification and analysis of gene sets that are differentially expressed in the D RAD**. We used fold change differences to obtain gene sets that are enhanced in the D RAD at each individual time point. The genes selected are at least three fold higher expressed in the D compared to the AR state and three fold higher expressed in the RAD compared to the MCE. The gene sets identified in the D RAD at 3, 7, 12 and 24 HAS, ranged from 11 to 30 genes in size. Underneath the mean $\log_2$ expression of each gene set is plotted in the D MCE (blue solid line), D RAD (blue dashed line) and the AR RAD (red dashed line). Under the graphs exemplar GO classes, found using ORA analysis, are indicated.

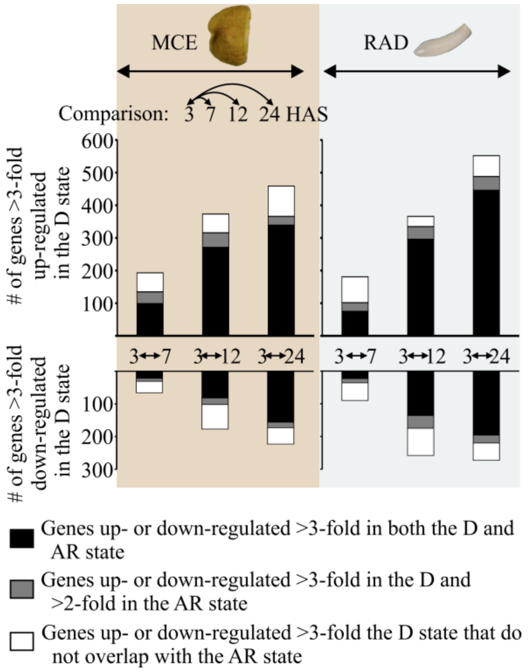


**Figure S4. Genes that are differentially expressed in D samples largely overlap with genes that are differentially expressed in AR samples.** The numbers of genes that are differentially expressed over 3-fold in the D MCE and RAD at successive time points (from 3-7, 3-12 and 3-24 HAS). Indicated are the number of genes that are also differentially expressed over three fold (in black) or over two fold (in grey) in AR samples. In white the number of genes that are not differentially expressed in the AR samples are shown.

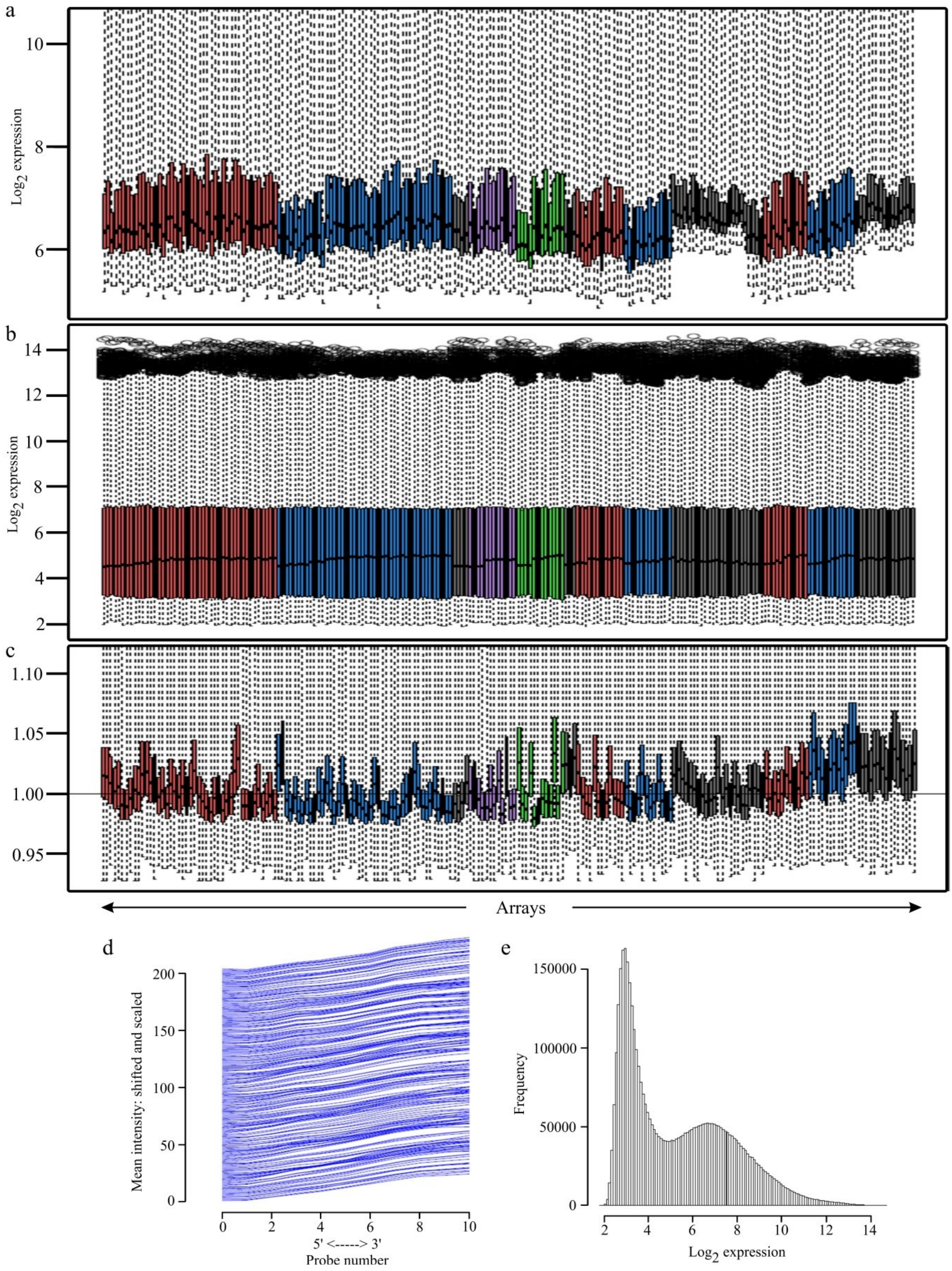


**Figure S5. Microarray quality assessment.** The data distributions before (a) and after normalization (b). After RMA normalization (Irizarry et al., 2003) the data distributions become comparable, although lower median values are found for the dry and shortly imbibed seeds, which are samples that were isolated from metabolically less active material. (c) Shows the Normalized Unscaled Standard Error (NUSE) of all arrays (d) RNA degradation plot shows comparable slopes for all arrays. (e) The histogram of the normalized data shows separated peaks for noise and signal, and the plot indicates a value of five (on a $log_2$ scale) as being potentially expressed.

**Table S1. Comparisons with other seed transcriptome datasets.** Several comparisons were made between our Cvi data set and various previously published data sets to investigate the overlap in differentially expressed found. In the first comparison it was investigated how many genes overlap with a dormancy and after-ripening gene list identified by Cadman et al. (2006). In the second comparison we identified genes that were either higher or lower expressed (using a 3-fold cut-off) between 3 and 24 HAS in the AR MCE (and RAD) in Cvi and investigated how many overlap in a similar comparison using the Col-0 germination data from Dekkers et al. (2013).

| Cadman et al. (2006) | # of genes in list [1] | # genes that overlap with the Cvi data set [2] |
|---|---|---|
| Dormancy list | 432 | **351 (81.3%)** |
| After-ripening list | 751 | **557 (74.2%)** |
| Cvi data set (this work) | # of differentially expressed genes comparing 24>3 HAS | # genes of the Col-0 data set (MCE or RAD 25N>MCE or RAD 3, Dekkers et al., 2013) which overlap |
| UP between 3-24 HAS in AR MCE | 1772 | **1268 (71.6%)** |
| UP between 3-24 HAS in AR RAD | 2082 | **1608 (77.2%)** |
| Cvi data set (this work) | # of differentially expressed genes comparing 3>24 HAS | # genes of the Col-0 data set (MCE or RAD3>MCE or RAD 25N, Dekkers et al., 2013) which overlap |
| DOWN between 3-24 HAS in AR MCE | 1238 | **870 (70.3%)** |
| DOWN between 3-24 HAS in AR RAD | 1517 | **1203 (79.3%)** |

[1] The number of genes from the Cadman et al. (2006) lists were used after removing any not present on our CustomCDF.

[2] The numbers were calculated using a 2-fold difference comparing D MCE 24>AR MCE 24 HAS and D RAD 24>AR RAD 24 HAS lists (and vice versa for AR).
